# A Novel Screening Approach for Familial Hypercholesterolemia: A Genetic Study on Patients Detected Using Preexisting Centralized Analytics

**DOI:** 10.3390/jcm14082780

**Published:** 2025-04-17

**Authors:** Joaquín Sánchez-Prieto, Fernando Sabatel, Fátima Moreno, Miguel A. Arias, Luis Rodríguez-Padial

**Affiliations:** Department of Cardiology, Toledo University Hospital, 45005 Toledo, Spain; fernando.sabatel.sspa@juntadeandalucia.es (F.S.); fatima.moreno.sanchez.sspa@juntadeandalucia.es (F.M.); maarias@sescam.jccm.es (M.A.A.); lrodriguez@sescam.org (L.R.-P.)

**Keywords:** familial hypercholesterolemia, cardiovascular disease, LDL receptor mutations

## Abstract

**Introduction and Objectives:** Familial hypercholesterolemia (FH) is an autosomal dominant genetic disorder of lipid metabolism that is characterized by elevated low-density lipoprotein cholesterol (LDL-C) levels and a high risk of atherosclerotic cardiovascular disease. Familial hypercholesterolemia is typically caused by mutations in the LDL receptor gene (LDLR), although other alterations may be found. The aim of this study was to perform a genetic study on a population identified through a new population-based diagnostic screen program for FH. **Methods:** Genetic variants in LDLR, apolipoprotein B (APOB), apolipoprotein E (APOE), proprotein convertase subtilisin/kexin type 9 (PCSK9), signal transducing Adaptor Family Member 1 (STAP1), low density lipoprotein receptor adaptor protein 1 (LDLRAP1) and lipase A, and lysosomal acid type lipase A (LIPA), as well as a genetic risk score, were evaluated in 84 individuals with a clinical diagnosis of FH based on the Dutch Lipid Clinics Network criteria (DLCN ≥ 6). These individuals were selected from a cohort of 752 patients with an abnormal lipid profile, obtained by screening existing centralized analytics. **Results:** A clinical diagnosis of FH was established in 17.9% of the patients evaluated, with mean LDL-C levels of 305.7 mg/dL (95% CI 250.4–360.9). Genetic variants were detected in 70.2% of these patients, with 50 different mutations identified, mainly in the LDLR. The most frequent pathogenic variants were c.1342C>T and c.313+1G>C. Null variants exhibited a more severe phenotype, and the risk score indicates that patients carrying genetic alterations have a 42% higher risk of developing cardiovascular disease. **Conclusions:** A high rate of genetic alterations was detected in patients with severe FH. In most cases, the phenotypic findings did not predict the genetic results, which provide important information regarding the cardiovascular risk of patients.

## 1. Introduction

Familial hypercholesterolemia (FH) is an autosomal dominant genetic disorder of cholesterol metabolism that is characterized by abnormally high levels of low-density lipoprotein cholesterol (LDL-C). FH confers an increased risk of premature atherosclerotic cardiovascular disease [1,2], with sudden death and ischemic heart disease being the main causes of mortality in this population [3]. Early diagnosis and treatment improve the prognosis of these patients [4], although recent evidence suggests that lipid-lowering treatments may often be insufficient, even in individuals with a history of ischemic heart disease [5,6]. FH is the most common cause of genetically derived cardiovascular disease [7] and while its prevalence has been estimated at 1:500 individuals, recent studies have raised this to 1:220 [8,9] or even higher in certain ethnic groups.

Despite being a genetic disorder, diagnosing FH is primarily based on clinical criteria [10,11]. Genetic testing confirms the definitive diagnosis and identifies the pathogenic variants associated with a higher cardiovascular risk that require intensive therapy [12]. However, genetic studies are essential to detect affected relatives early to ensure the prompt initiation of appropriate treatment. In fact, some experts recommend that genetic testing become standard for patients with probable or definite FH, as well as for their at-risk relatives [12].

A marked defect in the binding and internalization of LDL particles has been demonstrated in FH, usually caused by mutations in the gene encoding the LDL receptor (LDLR) _13_, which account for more than 95% of cases. Mutations in other genes involved in LDL uptake may also be detected, such as those in apolipoprotein B (APOB), proprotein convertase subtilisin/kexin type 9 (PCSK9) and low-density lipoprotein receptor adaptor protein 1 (LDLRAP1) [13]. However, in patients with an FH phenotype but no defined genetic alterations, other possible changes should be considered (e.g., polygenic inheritance, elevated lipoprotein, APOE variants or variants of as yet undescribed FH-related genes) [12].

There is currently only one national registry of FH patients in Spain that includes molecular data from them, information that will help in the early identification and treatment of new cases and their relatives [3,5]. This study examined a cohort of patients identified through a new population-based diagnostic screening for FH, including a genetic analysis with a view to enhancing our understanding of the phenotype/genotype relationships associated with FH that can help optimize treatments, and thereby improve prognoses.

## 2. Materials and Methods

### Patient Selection

This project is part of a study to evaluate the efficacy and efficiency of a new screening program to diagnose FH [14]. Using the central laboratory healthcare database for the region of Toledo (Spain), adult patients (>18 years old) whose lipid profiles met the inclusion criteria were selected (lipid profiles obtained through analyses performed for reasons unrelated to the study): total cholesterol >290 mg/dL, LDL-C >220 mg/dL, and triglycerides <200 mg/dL.

The study was conducted according to the guidelines of the Declaration of Helsinki, and approved by the Ethics Committee of Toledo University Hospital (protocol code 505P, June 2016).

A cohort of 752 patients was obtained from analyses carried out over the three years prior to the study, of whom 283 were excluded for different reasons: loss during follow-up, death, or concomitant pathologies that might affect the lipid values like hypothyroidism, hepatic, or pancreatic neoplasms, and other health conditions such as liver or kidney diseases that may affect the lipid profile.

The remaining 469 patients were evaluated clinically using the Dutch Lipid Clinics Network (DLCN) criteria, and they were classified as having possible FH (DLCN 3–5 points), probable FH (6–7 points), or definite FH (≥8 points). In addition to the DLCN criteria, anthropometric data, the presence of cardiovascular risk factors, a history of atherosclerotic disease (angina, myocardial infarction, or stroke), lipid profiles, renal and thyroid function, and details of prior and post-analysis lipid-lowering treatment (when available) were collected.

**Figure 1:**
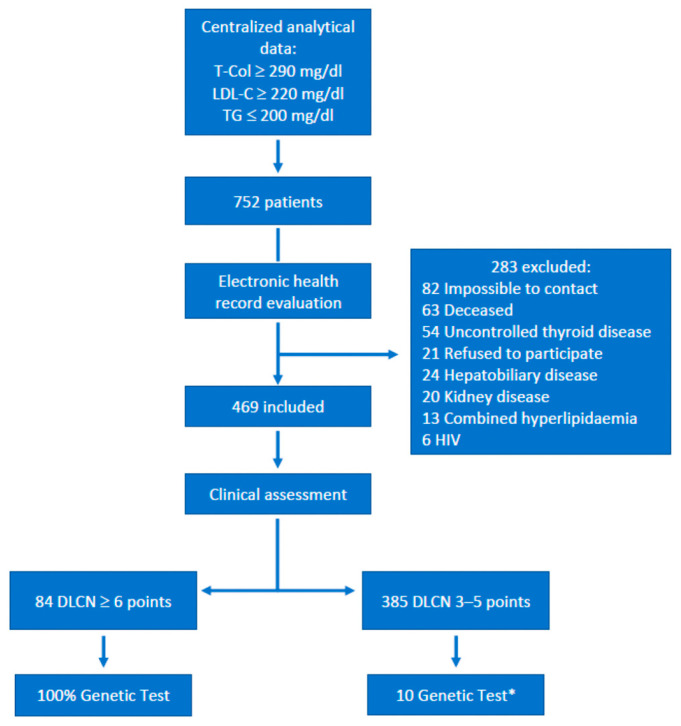
Patient selection. Flow chart explaining the steps for patient selection: * Selected by consecutive sampling to compare the proportion of positive genetic test results with a DLCN score ≥ 6: T-Col, total cholesterol; DLCN, Dutch Lipid Clinic Network criteria; LDL-C, low-density lipoprotein cholesterol; TG, triglycerides.

Patients with a probable or definite clinical diagnosis of FH (DLCN ≥ 6, *n* = 84, 17.9%), underwent genetic testing. To assess the accuracy of the genetic diagnosis in patients with possible FH, genetic testing was also performed on 10 randomly selected patients with a DLCN score of 3–5 points (Figure 1).

## 3. Genetic Analysis

Genetic testing was performed using the LIPID inCode^®^ test (GENinCode PLC, Oxford, UK), which has been used previously in Spanish studies [14,15]. This test is based on Next Generation Sequencing (NGS), and it involves capturing regions of interest by hybridization followed by ultra-sequencing. The results were analyzed using the Gendicall 3.0 bioinformatics tool, developed and validated by GENinCode [16]. The analysis detects alterations to the DNA sequence of the promoter, coding, and exon–intron junctions of seven genes: LDLR, APOB, PCSK9, APOE, STAP1, LDLRAP1, and LIPA [17,18,19,20]. Mutations of clinical relevance were classified as pathogenic, likely pathogenic, or of uncertain significance (Classes I, II, and III, respectively) [16]. Patients with mutations of uncertain significance were reclassified as pathogenic if they had more than two first-degree relatives with an FH phenotype, or when this familial information was not available or if the carrier had markedly elevated LDL-C (≥250 mg/dL) along with corneal arcus, age ≤ 45 years old, xanthomas, or premature atherosclerotic disease. A positive genetic diagnosis was established when an individual presented pathogenic, likely pathogenic or reclassified uncertain significance mutations. Those individuals with uncertain mutations that were not reclassified based on clinical data were considered to be negative. Finally, a genetic cardiovascular risk score (CARDIO inCode score) [21] was calculated for all the patients, which expresses the contribution of genetic variants (single nucleotide polymorphisms—SNPs) associated with atherosclerotic disease to cardiovascular risk as a ratio. This score reflects the weighted sum of the contribution of each genetic variant present in the individual, including those for FH, independent of those related to classical cardiovascular risk factors [22]. The score indicates the excess cardiovascular risk that can be attributed to the individuals genotype relative to the risk based on other classic factors.

## 4. Statistical Analysis

All the statistical analyses were performed using SAS software (version 9.3). Logistic regression was used to assess the associations between variables and Cohen’s d was calculated to estimate the standardized effect size. The frequencies of the qualitative variables were compared using a Chi-squared test and Fisher’s exact test. Pearson correlation was used to assess the associations between quantitative variables. Positive genetic values are expressed with their corresponding odds ratios (ORs). A *p*-value < 0.05 was considered statistically significant.

## 5. Results

A total of 469 patients from the initial 752 identified were evaluated clinically and their baseline characteristics were recorded (Table 1). Patients with a DLCN score ≥ 6 were significantly younger, had less hypertension, a higher prevalence of smoking, a more common history of personal and family cardiovascular disease, and more cases of severe hypercholesterolemia in first-degree relatives. This group also had higher LDL-C levels, and were treated more frequently and effectively (Table 1). Genetic data were obtained from all the patients with a probable or definite clinical diagnosis of FH (DLCN ≥ 6, *n* = 84), as well as from 10 patients with a possible FH diagnosis (DLCN 3–5). A positive result with the LIPID inCode^®^ genetic test was obtained from 67.9% of the patients with a DLCN score ≥ 6, with an OR of 8.44 (95% confidence interval [CI] 1.68–42.49). In patients with DLCN scores 6–7, corresponding to a probable FH diagnosis, 58.9% (33 patients) gave a positive genetic test result (OR 5.74, 95% CI 1.12–29.54 *p* = 0.037), whereas among the 28 patients with DLCN score ≥ 8, a definite diagnosis of FH, the genetic test gave a positive result in 24, 85.7% (OR 24.0, 95% CI 3.68–157 *p* = 0.001). By contrast, only 2 out of the 10 patients with a possible FH diagnosis (DLCN score 3–5 points) had a positive inCode genetic test result, identifying variants in LDLR and APOB.

### 5.1. Mutational Analysis

**Figure 2:**
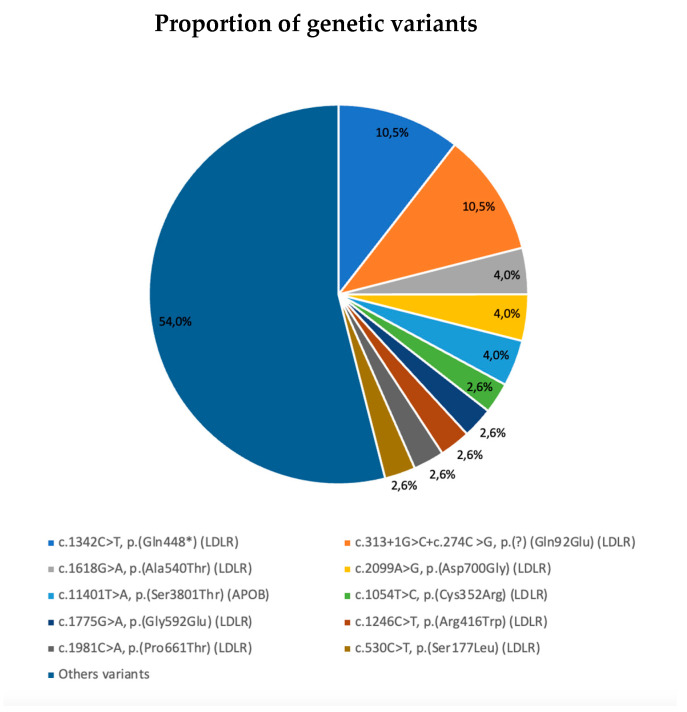
Proportion of the most frequent genetic variants in the cohort (*n* = 76).

Our genetic studies identified a total of 50 different mutations. Among the individuals with a DLCN score ≥ 6 that had a positive result in the genetic test, an isolated mutation was observed in 79.7% of cases, while combinations of mutations were detected in the remainder. The LDLR was that most frequently affected (89.8% of cases), either alone or in combination with other mutations. Following this, variants in the APOB gene were observed in 20.3% of the cases, whether isolated or in combination with others. By contrast only a small number of variants in the APOE, PCSK9, and LDLRAP1 genes were identified: an isolated mutation in APOE, one isolated mutation in PCSK9 and another in combination with an LDLR variant, and three mutations in LDLRAP1 in combination with LDLR variants. Indeed, the 10 most frequent mutations in our sample accounted for 46.0% of all the variants described (Table 2 and Figure 2).

Of the most frequent mutations, 21.0% have been classified as pathogenic, which included the two most commonly identified variants in our population. Conversely, up to 6.6% of the mutations most frequently described in our study have been classified as variants of uncertain significance, although these patients had markedly abnormal lipid profiles (LDL ≥ 250 mg/dL) and an affected first-degree relative.

Among those who underwent genetic testing, the average cardiovascular risk (Cardio inCode score) was 1.42, a 42% higher risk than when the same cohort was assessed with classic risk factors. In the patients diagnosed with FH, the Cardio inCode score was 1.31 as opposed to that of 1.56 in those patients not considered to suffer from FH, although this difference was not statistically significant.

### 5.2. Phenotype Versus Genotype

The patients were classified according to their allele type: null allele (*n* = 22, 37.3%), defective allele (*n* = 19, 32.2%), or not known (*n* = 18, 30.5%). They were also classified according to the type of mutation, as missense mutations (*n* = 32), nonsense mutations (*n* = 12), or other types of mutations (*n* = 15). Finally, they were categorized according to their pathology as Class I (*n* = 32, 54.2%), Class II (*n* = 17, 28.8%), or Class III (*n* = 10, 17%: Table 3). Notably, 20 of the 22 null alleles detected were identified in patients from pathogenic Class I. However, the remaining genetic variants detected were not related to any phenotypic findings. There was an association between the presence of null allele variants and a tendency toward higher LDL-C levels (an increase of +33.83 mg/dL: 95% CI −0.29 to 67.95, *p* = 0.052) and a higher DLCN score (+0.91 points: 95% CI −0.32 to 2.14 *p* = 0.147) relative to those with defective alleles, although these differences were not statistically significant.

The impact of the mutation type was assessed and nonsense mutations tended to be associated with higher LDL-C values than missense mutations (+15.82 mg/dL: 95% CI: −20.47 to 52.11 *p* = 0.395), albeit without reaching statistical significance. By contrast, intronic alternative splice mutations were associated with significantly higher LDL-C values (+50.43 mg/dL: 95% CI 9.99 to 90.88 *p* = 0.014) and higher DLCN scores (+2.52 points: 95% CI 1.13 to 3.91 *p* < 0.001) than nonsense mutations.

Pathogenic Class I showed a tendency to be associated with higher LDL-C values relative to Class II (+30.04 mg/dL: 95% CI −2.46 to 62.54, *p* = 0.070) and Class III (+31.49 mg/dL: 95% CI −7.74 to 70.72, *p* = 0.116). In terms of the DLCN scores, Class I was associated with significantly higher scores than Class II (+1.25 points: 95% CI 0.10 to 2.39, *p* = 0.038), although it was not significantly different to those associated with Class III (+1.35 points: 95% CI −0.03 to 2.73, *p* = 0.055). When comparing pathogenic Class I with the remainder of the available cases (*n* = 62), these patients were younger (44.2 as opposed to 50.21 years old, *p* = 0.035), and they had higher LDL-C values (317.0 mg/dL vs. 277.54 mg/dL, *p* = 0.0015) and higher DLCN scores (8.29 vs. 6.38, *p* < 0.001).

## 6. Discussion

This study presents some of the molecular characteristics of FH patients diagnosed through a new screening strategy in the local catchment area of the Toledo healthcare service. The patients were identified based on previously obtained LDL-C measurements, proven to be a feasible approach to rapidly detect cases warranting genetic testing. Our screening strategy presents several advantages that might justify the higher detection rates obtained. It focuses on patients with a higher probability of having FH by clinical diagnosis (DLCN ≥ 6), since this is the group that is actually considered to have FH and, therefore, are candidates for advanced lipid-lowering therapies in accordance with the recommendations of Spanish guidelines [23].

The most relevant findings were as follows: (1) the genetic test performed gave a positive result in a relatively high proportion of the patients with a DLCN score > 6; (2) in most cases only an isolated mutation was detected (79.7%), with the LDLR being affected in nearly 90% of the patients in which the genetic test gave a positive result; (3) most of the mutations found are considered to be pathogenic; (4) the risk scores obtained indicate that these patients have a 42% higher cardiovascular risk; and (5) the genetic variants detected were unable to distinguish between the specific phenotypes defined, except for the pathogenic Class I mutations. These results highlight the benefits of using genetic studies to complement the clinical diagnosis of FH, as molecular tools better characterize patients and provide an individualized cardiovascular risk assessment, potentially leading to improved treatment and prognosis [3].

The genetic alterations observed most frequently here were located in exon 9 and intron 3 of the LDLR (c.1342C>T and c.313+1G>C), constituting 21.04% of the mutations in our population. These two variants were also very prevalent in the Spanish SAFEHEART study, where they represented the first (7%) and third (5%) most common variants accounted for in that registry [24]. The effect of these alterations to the LDLR is well known, as they are consistently associated with premature ischemic heart disease [24]. By contrast, other Mediterranean populations exhibit a different mutational spectrum (e.g., in Greece), in which these variants are not predominant [25]. In that population, the c.1775G>A mutation in exon 12 was found to be the most frequent, as also described often in Germany and Italy [26]. However, in our registry this variant accounts for only 2.63% of the mutations and it was even less frequent in the SAFEHEART study. Other variants were seen to be more frequent in a Danish population [27], although the c.313+1G>C mutation was detected at a frequency similar to that observed in the Spanish population. These differences in the spectrum of variants among populations highlight the genetic heterogeneity of FH, with some variants being present in very few individuals and others occurring more frequently, although they may be fewer in number. Hence, population-based diagnostic protocols could potentially be tailored according to the known variant spectrum in a specific region.

Several polymorphisms were also detected when the exons of the genes studied were sequenced. Although these polymorphisms were not associated with the disease phenotype, it is possible that the presence and combination of multiple polymorphisms, even those yet to be recognized as functionally significant, might alter gene activity. This might generate a clinical and lipid profile similar to that observed in FH, a possibility that should be explored in future studies.

The genotype/phenotype associations in our sample, based on different allelic variants, provide valuable insights. In our cohort, null allele variants tended to exhibit higher total and LDL-C levels than defective variants, although this difference did not reach statistical significance probably due to the limited sample size. This observation aligns with other findings [28] and reinforces the idea that the molecular characterization of FH patients provides additional prognostic information, which would pave the way to the early initiation of more aggressive lipid-lowering therapies. Nonetheless, having determined the allele type, functional assays will be needed to determine if these alleles produce an LDLR with <2% activity. In our cohort, the proportion of null alleles was around 40%, somewhat lower than in other registries [29]. Gaining more accurate information about how different types of mutation influence cardiovascular risk would require a study of a heterogeneous cohort over an extended period, in which patients carrying the same mutation are included.

It is noteworthy that our cohort consists almost entirely of index cases, in contrast to other registers that include family cascade screening. This composition may make our sample optimal for a more detailed analysis of the genetic factors contributing to poor treatment responses and higher rates of cardiovascular disease.

In our series of patients, the prevalence of atherosclerotic disease in patients with FH established by molecular characterization (18.63%) was lower than that reported in some Western countries (30–39%) [30,31]. However, such comparisons are difficult as many earlier studies rely on a diagnosis of FH based solely on clinical criteria. We also believe that differences in the prevalence of cardiovascular disease may be influenced by other risk factors, as well as a possible modulatory effect of the Mediterranean diet (rich in monounsaturated fats), which has known cardiovascular benefits and antiatherogenic properties. The data from another Spanish registry, with a molecularly characterized population [24,32], is very similar to our cohort, with a prevalence of atherosclerotic disease (21.9%) closer to that seen here.

## 7. What Is New?

A new screening strategy for FH has been established, identifying a high proportion of positive genetic test results. This facilitates a rapid molecular characterization of the FH population and the quick identification of index cases. A 42% higher cardiovascular risk was documented in this population, yet the genetic variants detected do not distinguish specific phenotypes, except for the pathogenic Class I variants. The molecular study of FH patients may aid clinical decision-making and improve disease prognosis.

## 8. Conclusions

This study focused on the relationship between the molecular characteristics of FH patients diagnosed through a new screening program and their phenotype. The results highlight the genetic heterogeneity in this population, and they suggest that, on the whole, phenotype does not predict genetic results, although the latter may provide additional information regarding patient risk. Hence, it appears that the molecular characterization of FH patients can help establish a better prognosis, enabling prompt initiation of appropriate treatment to reduce LDL-C to recommended levels [33].

## Figures and Tables

**Table 1:** Baseline characteristics and the differences between patients with DLCN scores of 3–5 and ≥6.

Variables	Global (*n* = 469)	DLCN 3–5 (*n* = 385)	DLCN ≥ 6 (*n* = 84)	Standardized Effect Size (CI 95%) (a)
Men/Women	191 (40.7)/278 (59.3)	162 (42.1)/223 (57.9)	29 (34.5)/55 (65.5)	0.73 (0.44–1.19)
Age	53.2 ± 12.8	54.6 ± 12.3	47.1 ± 12.9	0.59 (0.36–0.82)
HTA	148 (31.6)	130 (33.8)	18 (21.4)	0.54 (0.31–0.94)
DM2	47 (10.0)	43 (11.2)	4 (4.8)	0.39 (0.14–1.14)
History of Smoking	233 (49.7)	181 (47.0)	52 (61.9)	1.83 (1.13–2.97)
Average BMI	27.7 ± 4.4	27.8 ± 4.3	27.0 ± 4.7	0.19 (−0.04–0.43)
Normal weight	135 (28.8)	104 (27.1)	31 (36.9)	1.57 (0.96–2.59)
Overweight	209 (44.6)	173 (45.1)	36 (42.9)	0.92 (0.57–1.47)
Obesity	124 (26.4)	107 (27.9)	17 (20.2)	0.66 (0.37–1.17)
Average DLCN score	4.25 ± 1.9	3.52 ± 0.8	7.56 ± 2.2	−2.07 (−2.21–−1.92)
Hypothyroidism	37 (7.9)	29 (7.5)	8 (9.5)	1.29 (0.57–2.94)
Chronic kidney disease	14 (3)	13 (3.4)	1 (1.2)	0.35 (0.04–2.67)
Cardiovascular disease	26 (5.5)	13 (3.4)	13 (15.5)	5.24 (2.33–11.77)
Family history of early ischemic heart disease	34 (7.2)	15 (3.9)	19 (22.6)	7.21 (3.49–14.91)
Family history of hypercholesterolemia	131 (27.9)	61 (15.9)	70 (83.3)	26.48 (14.02–49.99)
Corneal arcus (≤45 years)	7 (6.2)	0	7 (20.6)	-
Xanthomas	1 (0.2)	0	1 (1.1)	-
Total Cholesterol (mg/dL)	320.4 ± 25.3	316.1 ± 18.6	340.4 ± 38.9	−0.96 (−1.18–−0.74)
LDL-C (mg/dL)	238.3 ± 21.9	233.6 ± 13.6	259.8 ± 35.9	−1.19 (−1.40–−0.98)
HDL-C (mg/dL)	56.2 ± 14.0	56.4 ± 13.6	55.2 ± 15.9	0.08 (−0.15–0.33)
Triglycerides (mg/dL)	128.3 ± 36.3	129.5 ± 35.8	122.8 ± 38.4	0.18 (−0.05–0.42)
TSH (mlU/L)	2.1 ± 2.12	2.1 ± 2.3	2.2 ± 1.30	−0.08 (−0.32–0.17)
Hypolipidemic	344 (73.4)	269 (69.9)	75 (89.3)	3.59 (1.74–7.42)
High potency hypolipidemic	109 (23.2)	64 (16.6)	45 (53.6)	5.79 (3.49–9.59)

Values are mean ± standard deviation (SD) or *n* (%). * *p* < 0.05. ASCVD: atherosclerotic cardiovascular disease. CI: confidence interval. (a) Cohen’s d for continuous characteristics or odds ratio (OR) for categorical factors. Effects computed as the DLCN ≥ 6 cohort minus (or relative to) the DLCN 3–5 cohort. DLCN: Dutch Lipid Clinic Network. HCL: hypercholesterolemia. HDL-C: high-density lipoprotein cholesterol. LDL-C: low-density lipoprotein cholesterol. TSH: thyroid-stimulating hormone. * The reported effect sizes for some variables do not match the total sample size due to missing data.

**Table 2:** Description of the most frequent mutations in the registry.

cDNA	Protein	Gene	Exon	Effect	Pathogenic Class	Allele Type	N (%)
c.1342C>T,	p.(Gln448*)	LDLR (NM_000527.4)	9	Exonic-NonSense	Class I	Null	8/76 (10.5%)
c.313+1G>C+c.274C>G	p.(?) (Gln92Glu)	LDLR (NM_000527.4)	3i	Intronic-Splicing	Class I	Not available	8/76 (10.5%)
c.1618G>A	p.(Ala540Thr)	LDLR (NM_000527.4)	11	Exonic-Missense	Class II	Defective	3/76 (3.9%)
c.2099A>G	p.(Asp700Gly)	LDLR (NM_000527.4)	14	Exonic-Missense	Class II	Not available	3/76 (3.9%)
c.11401T>A	p.(Ser3801Thr)	APOB (NM_000384.2)	26	Exonic_Missense	Class III	Not available	3/76 (3.9%)
c.1054T>C	p.(Cys352Arg)	LDLR (NM_000527.4)	7	Exonic-Missense	Class II	Not available	2/76 (2.6%)
c.1775G>A	p.(Gly592Glu)	LDLR (NM_000527.4)	12	Exonic-Missense	Class I	Defective	2/76 (2.6%)
c.1246C>T	p.(Arg416Trp)	LDLR (NM_000527.4)	9	Exonic-Missense	Class I	Defective	2/76 (2.6%)
c.1981C>A	p.(Pro661Thr)	LDLR (NM_000527.4)	13	Exonic-Missense	Class III	Not available	2/76 (2.6%)
c.530C>T	p.(Ser177Leu)	LDLR (NM_000527.4)	4	Exonic-Missense	Class I	Null	2/76 (2.6%)

The 10 most frequent mutations in the FH cohort (*n* = 76) are shown, providing the following information: the cDNA alteration, the resulting protein, the gene, exon, the type of mutation, the pathogenic class, the allele type, and the number (percentage) of variants.

**Table 3:** Means and interquartile range of the lipid profile and DLCN score according to allele type, effect, and pathogenic class.

Allele Type	N	Total Cholest (mg/dL)/IQR	HDLC (mg/dL)/IQR	Triglycerides (mg/dL)/IQR	LDLC (mg/dL)/IQR	DLCN (points)/IQR
Defective	19	339 (291.1–386.9)	53.1 (42.9–63.2)	110.9 (71.5–150.3)	287.6 (244.2–330.9)	7.3 (5.0–9.6)
Not available	18	337.4 (302.1–372.6)	53.2 (38.2–68.3)	128.7 (90.4–167.0)	305.6 (253.1–358.1)	7.3 (6.1–8.6)
Null	22	355.7 (312.0–399.4)	52.9 (36.2–69.7)	126.8 (86.1–167.5)	321.4 (257.8–385.0)	8.2 (6.1–10.3)
Effect						
Exonic-Del	3	400.0 (377.3–422.7)	46.7 (42.1–51.3)	147.3 (95.3–199.4)	363.3 (288.8–437.9)	8 (6.3–9.7)
Exonic-Deletion-InFrame	1	442	99	118	319	6
Exonic-Synonymous	1	382	49	190	295	7
Intronic-Splicing	9	347.1 (295.0–399.2)	48.1 (41.2–55.0)	112.0 (70.0–153.9)	340.8 (292.3–389.2)	9.5 (7.5–11.6)
Missense	32	334.6 (297.6–371.6)	53.4 (42.4–64.3)	117.1 (83.1–151.2)	290.3 (240.8–339.9)	7.0 (5.5–8.6)
Nonsense	12	345.8 (305.3–386.3)	53.2 (32.2–74.3)	126.0 (80.9–171.1)	306.2 (244.1–368.2)	8.1 (5.7–10.6)
Promoter	1	333	63	195	300	7
Pathogenic class						
Class I	32	351.1 (300.0–402.1)	53.0 (37.1–69.0)	117.7 (79.2–156.2)	319.7 (260.7–378.7)	8.2 (5.9–10.6)
Class II	17	335.5 (307.5–363.5)	53.9 (43.5–64.3)	107.9 (73.8–141.9)	289.6 (238.3–340.9)	7.0 (5.9–8.1)
Class III	10	340.0 (306.0–374.0)	51.8 (37.2–66.3)	161.2 (132.1–190.3)	288.2 (249.5–236.9)	6.9 (5.9–7.9)

## Data Availability

Data are contained within the article.
